# Adherence, satisfaction and functional health status among patients with multiple sclerosis using the BETACONNECT® autoinjector: a prospective observational cohort study

**DOI:** 10.1186/s12883-017-0953-8

**Published:** 2017-09-06

**Authors:** Ingo Kleiter, Michael Lang, Judith Jeske, Christiane Norenberg, Barbara Stollfuß, Markus Schürks

**Affiliations:** 1 St. Josef Hospital, University Hospital Bochum, Bochum, Germany; 2 Present Address: Marianne-Strauß-Klinik, Behandlungszentrum Kempfenhausen für Multiple Sklerose Kranke, Berg, Germany; 3 Joint Neurological Practice, Ulm, Germany; 4 Neurological Practice, Wuppertal, Germany; 5 Bayer AG, Wuppertal, Germany; 6 Bayer Vital GmbH, Leverkusen, Germany

**Keywords:** Multiple sclerosis, Disease modifying drugs, Interferon beta-1b, Autoinjector, BETACONNECT®, Adherence, Persistence, Compliance, Satisfaction

## Abstract

**Background:**

Maintaining patient adherence to disease modifying drugs in multiple sclerosis is a challenge, which can be improved by autoinjectors. The BETACONNECT® is a fully electronic autoinjector for the injection of interferon beta-1b (IFN beta-1b) automatically recording injections.

**Methods:**

The BETAEVAL study was a prospective, observational, cohort study over 24 weeks among patients with relapsing remitting multiple sclerosis or clinically isolated syndrome treated with IFN beta-1b in Germany using the BETACONNECT®. The primary aim was to investigate treatment adherence, secondary aims included assessing satisfaction and functional health status. Adherence was evaluated from injection data recorded by the device. Patient-related data were obtained from clinical examinations and patient questionnaires.

**Results:**

Of the 151 patients enrolled, 143 were available for analysis. Thirty-four patients discontinued the study prematurely. 107/143 (74.8%) patients still used the BETACONNECT® at the end of the study. Injection data from the device at any visit was available for 107 patients. Among those, the percentage of adherent patients injecting ≥80% of doses and still participating in the study was 57.9% at week 24. 29% of patients prematurely stopped the study, 13.1% injected <80%. Among patients with BETACONNECT® data at the respective visit, the proportion of adherent patients was high over the entire study period (week 4: 81.1% [*N* = 95], week 12: 86.7% [*N* = 83], week 24: 80.5% [*N* = 77]). Participants (*N* = 143) indicated high satisfaction with the BETACONNECT®. At week 24, 98.0% of patients who completed the corresponding questionnaire (strongly) agreed that it was user-friendly, 81.2% felt confident in using it compared to their previous way and 85.5% preferred it to their previous way of injection. Injection-related pain was rated as mild to moderate at all follow-up visits. Whereas 17.2% of patients with corresponding questionnaire indicated using analgesics prior to injection at week 4, only 9.1% did at week 24. Outcomes from questionnaires assessing functional health status, depression, fatigue and cognitive function were very similar throughout the study course.

**Conclusions:**

The majority of patients continued using the BETACONNECT® for IFN beta-1b treatment during the 24-week study period. Adherence was high among participants still using the BETACONNECT® and patients were highly satisfied with the device. Ongoing studies will evaluate long-term adherence and treatment outcomes in patients using the BETACONNECT®.

**Trial registration:**

clinicaltrails.gov NCT02121444 (registered April 22, 2014).

**Electronic supplementary material:**

The online version of this article (10.1186/s12883-017-0953-8) contains supplementary material, which is available to authorized users.

## Background

Multiple sclerosis (MS) is a chronic inflammatory and degenerative autoimmune disease of the central nervous system affecting an estimated 200,000 patients in Germany [1]. To date, there is no cure for MS and current treatment strategies aim at reducing relapses and slowing disease progression. Interferon beta and glatiramer acetate are established and well-characterized disease modifying drugs (DMDs) for the treatment of MS, that are administered by subcutaneous (sc) or intramuscular (im) injections. Long-term adherence to the prescribed treatment regimen is crucial in order to achieve optimal response from injectable DMDs. Patients missing doses or interrupting treatment fare worse and have an increased risk of relapse and disability progression than adherent patients [2–5]. This directly translates into increased healthcare resource utilization which may lead to increased healthcare costs [4, 5]. However, adherence was found to be suboptimal in available studies of injectable DMDs [3, 5–7].

Thus, improving adherence to DMDs in MS remains a challenge and requires new strategies to help patients overcome potential barriers and maintain their injection schemes. These strategies include patient education, adequate management of side effects, improvement in drug formulation and drug delivery devices [8]. Use of autoinjectors may improve tolerability of injections for example by reducing local skin reactions [8, 9], and was found to be a strong predictor of adherence at 24 months in an observational study [10]. The development of autoinjectors is ongoing in order to further facilitate the injection process for patients [10]. The new BETACONNECT® is a fully electronic autoinjector for the subcutaneous administration of interferon beta-1b (IFN beta-1) applying a four-phase injection technology as described previously [11]. It was designed to further improve handling and allows choosing of individual injection settings, such as injection speed and depth. In addition, it offers an electronic reminder function. Injection-related information such as injection date and time, injection depth, speed and volume are automatically stored in the device.

Here we present the results of the observational BETAEVAL study investigating adherence and satisfaction of MS patients using the BETACONNECT® autoinjector in a real-world setting in Germany over 24 weeks.

## Methods

### Study design and participants

The BETAEVAL study (NCT02121444) was a prospective, non-interventional, observational cohort study in Germany sponsored by Bayer Vital GmbH. Patients were consecutively enrolled in 35 neurological offices and clinics specializing in the treatment of MS patients and followed for 24 weeks. The study was performed between June 2014 and March 2016. Visits were done at baseline, 4, 12 and 24 weeks. All participants provided written informed consent. All treatment decisions, including the decision on treatment with Betaferon® was made by the attending physicians.

### Eligibility

Patients with relapsing remitting multiple sclerosis (RRMS) or with a clinically isolated syndrome (CIS), who were treated or were starting treatment with IFN beta-1b and agreed to use the BETACONNECT® autoinjector, were eligible for participation. Exclusion criteria were treatment with other DMDs or contraindications to IFN beta-1b as stated in the prescribing information [12].

### Objectives

The primary objective was to investigate adherence to therapy among patients treated with IFN beta-1b using the BETACONNECT® autoinjector. Secondary objectives included investigation of satisfaction with and evaluation of the BETACONNECT® autoinjector. Furthermore, injection site pain, prophylactic analgesic use, and local skin reaction as well as depression and anxiety, health-related quality of life, fatigue and cognition were assessed.

### Definitions of the primary outcome variables

The BETACONNECT® automatically records injections. During the time of observation, the number of injections recorded was compared to the number of expected injections. Patients injecting ≥80% of the expected dosages were considered adherent. Adherence was assessed for different patient populations:

1. Adherence among all patients with at least one data readout from BETACONNECT®: percentage of patients injecting ≥80% of the prescribed dosages among those with at least one BETACONNECT® readout. Patients prematurely discontinuing the study before a certain visit were considered non-adherent at that visit and at all subsequent visits.
2. Adherence among patients with BETACONNECT® readings at corresponding visit: percentage of patients injecting ≥80% of the prescribed dosages among those with BETACONNECT® readouts at the respective visit.

Persistence to the BETACONNECT® device was defined as the percentage of patients still using the BETACONNECT® autoinjector at each follow-up visit.

Compliance among patients with data from BETACONNECT® was defined as the percentage of prescribed injections applied within the observational period and calculated as follows: compliance (%) = ((documented no. of treatment days during observation)/ (expected no. of treatment days during observation period)) ×100.

### Training of study investigators

Study investigators were obliged to attend an online training presentation prior to enrolment of patients. The training provided detailed information about the aims and course of the study as well as the process of electronic data documentation. After passing a test and answering specific questions, investigators were allowed to document patients.

### Data collection and analysis

All data were entered into electronic case report forms (eCRFs) and saved in an electronic data capture system (EDC system).

During the study period, the investigators documented demographic data (age, gender, employment status, educational level), medical history (including history of MS and prior therapy), participation in the BETAPLUS® nurse support programme, and disease-related variables based on the results of detailed clinical examinations and tests, including local skin reactions, expanded disability status scale (EDSS) [13], and Symbol Digit Modalities Test (SDMT) [14]. The SDMT is used to screen for cognitive dysfunction and measures attention, concentration and information processing speed. In the substitution task, the examinee is given 90 s to pair specific numbers with given geometric figures.

Injection data were automatically recorded by the BETACONNECT® on every injection. Investigators were asked to download data from the device during scheduled visits and upload them into the eCRF.

Further data were obtained from patients via questionnaires completed during site visits. Patient satisfaction with and evaluation of the BETACONNECT® autoinjector as well as injection site pain and prophylactic analgesic use were assessed with a questionnaire developed for the BETAEVAL study (see Additional file 1). Other patient-reported outcomes were documented using the following rating scales: functional assessment of multiple sclerosis (FAMS) [15], Hospital Anxiety and Depression Scale (HADS) [16], Centre for Epidemiologic Studies Depression Scale (CES-D) [17], Fatigue Scale for Motor and Cognitive Functions (FSMC) [18]. A detailed description of these scales is provided in the Additional file 2.

The completed questionnaires were collected by the investigator and sent to the CRO, where the data were double-entered into the eCRF.

Patients with available data at baseline as well as at least one post-baseline visit were included in the full analysis set (FAS).

### Statistical analysis

Statistical analysis was performed with SAS Version 9.3. Statistical analyses were exploratory and descriptive in nature using mean (± standard deviation [SD]), median, minimum, maximum) for continuous variables, and category counts and frequencies (percentages) for categorical variables.

Logistic regression employing a stepwise selection procedure was used to investigate baseline predictors of adherence and persistence. The entry level was *p* = 0.5 and the stay level *p* = 0.1. All covariates being still nominally significant were considered as associated to adherence/persistence.

The following baseline covariates were considered in the prediction model: age (years), baseline EDSS score (<3, ≥3), CES-D at initial visit (<16, 16–21, >21), concomitant diseases (yes, no), concomitant medication (yes, no), education level (elementary school, secondary school, apprenticeship, college/university), total FAMS at initial visit (0–176), FSMC at initial visit (<43, 43- < 53, 53- < 63, ≥63), gender (female, male), HADS (anxiety) at initial visit (<8, ≥8), MS duration (months), participation in BETAPLUS® (yes, no), previous treatment (none, Betaferon®, other than Betaferon®), pain intensity with previous way of Betaferon® injection (<4, ≥4, no previous Betaferon® intake), SDMT at initial visit (0–110), and type of previous Betaferon® injection (none, manual, autoinjector).

For primary outcome variables, statistical analysis was based on the available data only and missing data were not imputed.

Questionnaires were scored according to standard rules based on available instructions. For the regression models in secondary analyses missing values in the questionnaire scores were either replaced by the mean or median of the available values (continuous data) or a separate category was created (categorical data).

### Safety

All patients who took at least one dose of Betaferon® and provided sufficient information as to whether they experienced an adverse event or not were included in the safety set (SAF).

Adverse events (AEs) and device events (including handling errors) were documented during the whole observation period.

## Results

### Patient disposition

**Fig. 1:**
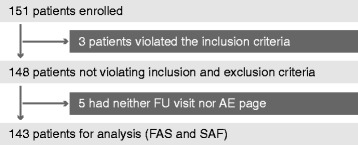
Flow chart describing patient disposition in the BETAEVAL study. *FU* follow-up; *AE* adverse events; *FAS* full analysis set; *SAF* safety analysis set

The BETAEVAL study consecutively enrolled 151 patients. Of those, 8 patients were excluded because the inclusion criteria were violated or no documented follow-up visit was available, resulting in 143 patients for final analysis (full analysis set (FAS) and safety analysis set (SAF); Fig. 1).

### Baseline demographics and characteristics

The demographic and clinical characteristics of the patient population at the time of enrolment are summarized in Table 1. The median age was 40 (range: 21–79) years and about two thirds of the patients (69.2%) were women. Most patients were diagnosed with RRMS (95.1%), 4.9% with CIS. The median duration from the initial event until time of diagnosis was 1.32 months (range 0–137.3), the median duration of disease was 29.9 months (range 0–372.6) and the median EDSS at baseline was 2.0 (0–6.5). Almost three quarters of patients (74.1%) had received Betaferon® previously and among those, 79.3% were experienced in using an autoinjector. 60.8% of all patients participated in the BETAPLUS® nurse support programme.

**Table 1:** Baseline demographics and clinical characteristics among participants of the BETAEVAL study (*N* = 143)

Characteristic	
Age, years	
Mean (SD)	41.2 (11.5)
Median (range)	40 (21–79)
Gender, n (%)	
Women	99 (69.2)
Men	44 (30.8)
BMI, kg/m^2^	
Mean (SD)	25.8 (5.2)
Median (range)	24.5 (17.6–47.8)
Diagnosis, n (%)	
RRMS	136 (95.1)
CIS	7 (4.9)
Time between first symptoms and initial diagnosis, months; (*N* = 87)	
Mean (SD)	15.2 (29.4)
Median (range)	1.32 (0–137.3)
Duration of disease, months; (*N* = 105)	
Mean (SD)	57.5 (73.0)
Median (range)	29.9 (0.0–372.6)
EDSS, median (range); (*N* = 124)	2.0 (0–6.5)
Previous treatment, n (%)	
Betaferon®	106 (74.1)
Other treatment	6 (4.2)
No previous treatment	31 (21.7)
Previous usage of auto-injector for Betaferon® treatment among patients who received Betaferon® previously (*N* = 106), n (%)	
Any	84 (79.3)
BETACOMFORT®	51 (60.7)
BETAJECT Comfort®	20 (23.8)
BETAJECT lite®	3 (3.6)
Other	10 (11.9)
Participation in BETAPLUS® nurse support programme, n (%)	87 (60.8)
Employment status, n (%)	
Employed	107 (74.8)
Retired	13 (9.1)
Keeping house	7 (4.9)
Student	3 (2.1)
Seeking work	3 (2.1)
Self-employed	2 (1.4)
Other	3 (2.1)

Numbers (%) may not add up to total numbers (100%) due to missing values

*SD* standard deviation, *BMI* body mass index, *RRMS* relapsing remitting multiple sclerosis, *CIS* clinically isolated syndrome, *EDSS* expanded disability status scale

### Persistence, adherence and compliance

Among the 143 patients in the FAS, 129 (90.2%) indicated they were still performing their injections with the BETACONNECT® at week 4, 123 (86%) at week 12 and 107 (74.8%) at week 24. Table 2 provides a stratified analysis of persistence for specific subgroups of patients. Persistence in using the BETACONNECT® at week 24 was numerically higher among patients ≥40 years, men, and patients with a baseline EDSS <3. Conversely, patients previously treated with IFN beta-1b and patients participating in the BETAPLUS programme were less persistent in using the device than treatment-naïve patients or patients without BETAPLUS participation.

**Table 2:** Persistence, adherence, and compliance stratified by age, gender, baseline EDSS score, and participation in BETAPLUS programme

	Week 4		Week 12		Week 24	
Number of persistent patients (%)						
Total (FAS, *N* = 143)	90.2		86.0		74.8	
Age						
< 40 (*N* = 69)	89.9		84.1		69.6	
≥ 40 (*N* = 74)	90.5		87.8		79.7	
Gender						
Female (*N* = 99)	90.9		83.8		73.7	
Male (*N* = 44)	88.6		90.9		77.3	
EDSS baseline score						
< 3 (*N* = 90)	92.2		90		78.9	
≥ 3 (*N* = 34)	91.2		85.3		67.7	
Previous treatment with IFN beta-1b						
Yes (*N* = 106)	89.6		84.0		70.8	
No (*N* = 37)	91.9		91.9		86.5	
BETAPLUS participation						
Yes (*N* = 87)	87.4		81.6		70.1	
No (*N* = 56)	94.6		92.9		82.1	
Number of adherent patients (%) among all patients with at least one data readout from Betaconnect						
Total (*N* = 107)	72.0		67.3		58.0	
Age						
< 40 (*N* = 46)	71.7		65.2		56.5	
≥ 40 (*N* = 61)	72.1		68.		59.0	
Gender						
Female (*N* = 75)	74.7		66.7		57.3	
Male (*N* = 32)	65.6		68.8		59.4	
EDSS baseline score						
< 3 (*N* = 67)	76.1		71.6		58.2	
≥ 3 (*N* = 27)	63.0		55.6		55.6	
Previous treatment with IFN beta-1b						
Yes (*N* = 75)	68.0		62.7		49.3	
No (*N* = 32)	81.3		78.1		78.1	
BETAPLUS participation						
Yes (*N* = 69)	76.8		69.6		59.4	
No (*N* = 38)	63.2		63.2		55.3	
Number of adherent patients (%) among all patients with data from corresponding visit						
	*N*	*%*	*N*	*%*	*N*	*%*
Total	95	81.1	83	86.8	77	80.5
Age						
< 40 years	40	82.5	34	88.2	30	86.7
≥ 40	55	80	49	85.7	47	76.6
Gender						
Female	66	84.9	57	87.7	53	81.1
Male	29	72.4	26	84.6	24	79.2
EDSS baseline score						
< 3	59	86.4	54	88.9	47	83.0
≥ 3	24	70.8	18	83.33	19	79.0
Previous treatment with IFN beta-1b						
Yes	66	77.3	56	83.9	50	74.0
No	29	89.7	27	92.6	27	92.6
BETAPLUS participation						
Yes	61	86.9	53	90.6	49	83.7
No	34	70.6	30	80.0	28	75.0
Mean compliance (%) among all patients with data from corresponding visit						
Total	95	86.3	83	91.9	77	92.9
Age						
< 40 years	40	87.1	34	93.2	30	94.8
≥ 40	55	85.2	49	91.0	47	91.6
Gender						
Female	66	88.8	57	91.5	53	93.47
Male	29	80.7	26	92.8	24	91.6
EDSS baseline score						
< 3	59	89.72	54	93.5	47	94.9
≥ 3	24	79.4	18	89.4	19	90.4
Previous treatment with IFN beta-1b						
Yes	66	84.5	56	91.1	50	90.2
No	29	90.4	27	93.4	27	97.8
BETAPLUS participation						
Yes	61	91.2	53	93.4	49	93.9
No	34	77.6	30	89.1	28	91.0

*FAS* full analysis set, *EDSS* expanded disability status scale, *IFN* interferon

Age tends to be a predictor of persistence at 24 weeks with patients ≥40 years being more likely to still use the BETACONNECT® at follow-up visits (odds ratio [OR] 1.047, 95-%-confidence interval [CI]: 1.003–1.093). Similarly, a higher persistence was observed in patients who were naïve to IFN beta-1b treatment (OR = 12.246, 95-%-CI: 2.191–68.457).

Injection data from the BETACONNECT® were not available for all patients and for some patients not at all visits. At least one data readout from the device at any visit was available for *N* = 107 patients. The number of patients with available injection data at a visit was *N* = 95 (week 4), *N* = 83 (week 12) and *N* = 77 (week 24).

**Fig. 2:**
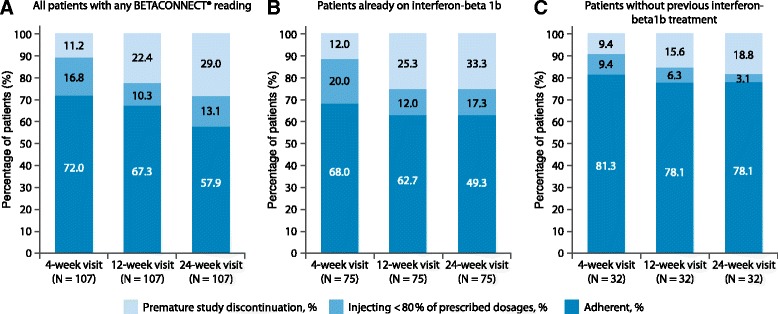
**a**: all patients with any BETACONNECT® reading; **b**: patients already on interferon-beta 1b; **c**: patients without previous interferon-beta 1b treatment

Among the group of patients with at least one data readout from the BETACONNECT, adherence declined from 72.0% at week 4 to 67.3% at week 12 and 57.9% at week 24. The percentage of patients still using the BETACONNECT® but injecting less than 80% of the prescribed dosages ranged between 16.8% (week 4) and 10.3% (week 12). Thus, the decline in adherence was mainly driven by premature study discontinuation (11.2% at week 4, 22.4% at week 12 and 29% at week 24) (Fig. 2a). Results from stratified analysis of adherence among patients with at least one readout from BETACONNECT® are provided in Table 2. Adherence was numerically higher in patients without prior treatment with IFN beta-1b and in patients participating in the BETAPLUS programme than in the corresponding comparison group at all visits. Moreover, while adherence declined among patients experienced with IFN beta-1b (Fig. 2b), it remained stable among those previously untreated (week 4: 81.3%, week 12: 78.1% and week 24: 78.1%) (Fig. 2c). Accordingly, being newly treated with IFN-beta-1b was a positive predictor for adherence (OR = 5.647, 95-%-CI: 1.775–17.969).

**Fig. 3:**
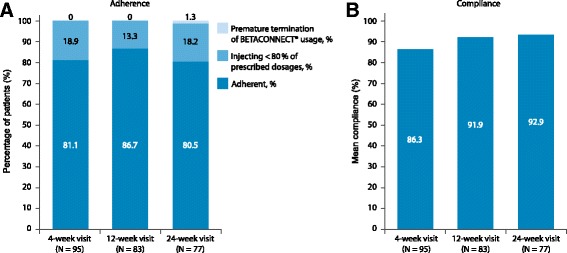
**a** Adherence and **b**: compliance (percentage of injections administered) among patients with BETACONNECT® data at respective visit

Among patients with available data from the BETACONNECT® at the respective visit, adherence remained stable and more than 80% of the patients injected ≥80% of their prescribed doses throughout the study period (week 4: 81.1%; week 12: 86.7%; week 24: 80.5%; Fig. 3a). At the 24-week visit, one patient indicated having stopped using BETACONNECT prior to the 24-week visit, but handed in his device for data readout.

Similarly, compliance remained high throughout the study among patients with available data from the BETACONNECT® at the respective visit. At week 4, on average 86.3% of the prescribed doses were taken with a slight increase to 91.9% at week 12 and 92.9% at week 24 (Fig. 3b).

Stratified analyses for both adherence and compliance among patients with BETACONNECT® readout at the respective visit are presented in Table 2. Logistic regression analysis did not identify predictors for these two outcome variables.

### Satisfaction with and evaluation of the BETACONNECT®

Satisfaction with and evaluation of the BETACONNECT® were assessed in the full analysis set (*N* = 143). Satisfaction with the BETACONNECT® was very high throughout the study duration. On a scale from 0 to 10, mean satisfaction with the previous way of Betaferon® injection was 7.4 and ranged between 8.3 and 8.5 with the BETACONNECT® (Table 3). The high level of satisfaction with the BETACONNECT® was very similar in subgroups after stratification by age, gender, baseline EDSS, previous IFN beta-1b treatment or participation in the BETAPLUS programme (Additional file 3: Table S1).

**Table 3:** Satisfaction with the BETACONNECT

Question	Initial visit	Follow-up visit after 4 weeks	Follow-up visit after 12 weeks	Follow-up visit after 24 weeks
	…way of injection before study start	…BETACONNECT®		
	*N* = 93	*N* = 115	*N* = 109	*N* = 99
Satisfaction with…, NAS range 0–10				
Mean (SD)	7.4 (2.0)	8.3 (1.9)	8.5 (1.5)	8.4 (1.5)
Median (range)	8 (1–10)	9 (1–10)	9 (2–10)	9 (3–10)
	*N* = 94	*N* = 114	*N* = 110	*N* = 98
Injection related pain with…, NAS range 0–10				
Mean (SD)	4.2 (2.5)	3.6 (2.5)	4.0 (2.6)	4.1 (2.6)
Median (range)	4 (0–10)	3 (0–10)	4 (0–10)	4 (0–10)
	*N* = 96	*N* = 116	*N* = 111	*N* = 99
Prophylactic use of analgesics prior to injection with…, % (patients with corresponding questionnaire)	14.6	17.2	17.1	9.1

*NAS* numerical analogue scale; *SD* standard deviation

**Fig. 4:**
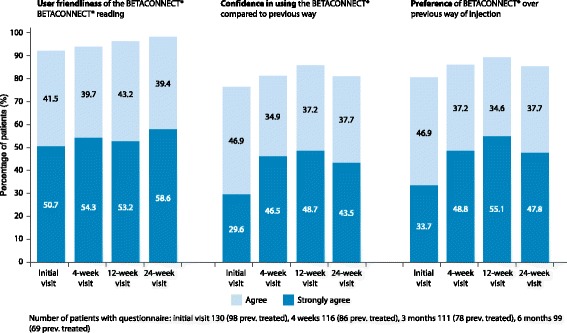
Evaluation of the BETACONNECT®

The vast majority of patients agreed or strongly agreed that the BETACONNECT® was user-friendly. This proportion increased in patients with corresponding questionnaire from 92.3% at the initial visit (120 of 130 patients) to 98.0% at 24 weeks (97 of 99 patients). Considerably fewer patients (80.2%; 77 of 96 patients) held this opinion of their previous way of injection. 76.5% (75 of 98 patients) of patients with corresponding questionnaire indicated that they felt confident in using the BETACONNECT compared to the previous way at the initial visit, rising to 85.9% (67 of 78 patients) at 12 weeks and 81.2% (56 of 69 patients) at 24 weeks (Fig. 4). Consistently, an increasing proportion of patients preferred the BETACONNECT® over their previous way of injection (initial visit: 80.6%; 79 of 98 patients, week 24: 85.5%; 59 of 69 patients) (Fig. 4). The proportion of patients strongly agreeing to these questions was higher at the follow-up visits than at the initial visit.

Throughout the study injection-related pain was rated as mild to moderate for the BETACONNECT® as well as for the previous way of injection. On a scale from 0 to 10, the median pain intensity was 3 (week 4) and 4 (week 12 and 24) with the BETACONNECT® and 4 with the previous way of injection (Table 3). While 14.6% of patients with corresponding questionnaire used analgesics prophylactically before the study start, 17.2% did so at week 4 with the BETACONNECT, 17.1% at week 12, and only 9.1% at week 24 (Table 3).

Analyses stratified by age, gender, baseline EDSS, previous IFN beta-1b treatment or participation in the BETAPLUS programme indicated subgroup-specific differences with respect to injection-related pain and use of analgesics. Median injection related pain at week 24 (range) was: age < 40: 5 (0, 9.5), age ≥ 40: 3 (0, 10); women: 4 (0, 10), men: 3 (0, 8); previous IFN beta-1b treatment: 3 (0, 10), no previous IFN beta-1b treatment: 5 (0, 9); participation in BETAPLUS programme: 4 (0, 9), no participation in BETAPLUS programme: 3 (0, 10). No difference in median injection-related pain was seen for baseline EDSS: EDSS <3: 4 (0, 10), EDSS ≥3: 4 (0, 9). Use of analgesics at week 24 (patients with corresponding questionnaire): age < 40: 16.3%, age ≥ 40: 3.6%; women: 11.9%, men: 3.1%; baseline EDSS <3: 9.4%, baseline EDSS ≥3: 12.5%; previous IFN beta-1b treatment: 5.8%, no previous IFN-beta-1b treatment: 16.7%. The use of analgesics was similar in patients participating or not participating in the BETAPLUS programme (8.9% and 9.3%, respectively). However, in all subgroups except patients <40 years, the proportion of patients using analgesics was lower at the end of the study than at the study start. Detailed stratification of injection related pain and the use of analgesics is provided in Additional file 4: Table S2 and Additional file 5: Table S3.

### Local skin reactions

Local skin reactions were reported in 16.3% of patients at week 4, 14.4% at week 12 and 12.8% at week 24. Redness was the most frequently reported skin reaction (week 4: 80.8%, week 12: 85.7%, week 24: 77.8% of reported reactions). Haematoma and induration were occasionally observed (haematoma: week 4: 2 cases. Induration: week 4: 2 cases, week 12: 2 cases, week 24: 4 cases). The reactions were mainly classified as mild (almost 75%) or moderate.

### Patient-related measures

Only 5 patients (3.5%) experienced further demyelinating events/relapses during the study, corresponding to an annualised relapse rate of 0.09.

Patient-related outcomes from questionnaires evaluating health-related quality of life, depression, anxiety, fatigue, as well as results from the cognition assessment were very similar at the beginning and at the end of the study (Table 4). Stratification of the mean scores for age, gender, EDSS baseline score, previous treatment with IFN-beta-1b and participation in the BETAPLUS programme is provided in Additional file 6: Table S4. Scores were rather similar across strata except for patients with an EDSS baseline score ≥ 3, who scored higher on the HADS anxiety and depression scale, CES-D and FSMC scales (Additional file 6: Table S4).

**Table 4:** Patient-related outcome measures

Questionnaire	Initial visit	24-week visit	Results from other published studies in patients with MS	
FAMS (scale: 0–176) Total score (without regard to additional concerns)	*N* = 129	*N* = 97		
Mean (SD)	135.5 (29.2)	134.6 (33.7)	120.3 (33.2)	*N* = 137 [19]
			120.3 (30.7)	*N* = 344 [20]
			107.5 (32.9)	*N* = 377 [15]
			110.4 (31.7)	*N* = 463–472 [21]
Median (range)	145.2 (58–171)	146 (56–176)	----	
HADS – anxiety (scale: 0–21)	*N* = 131	*N* = 94		
Mean (SD)	5.3 (4.1)	5.4 (4.3)	8.03 (4.2)	*N* = 4516 [23]
Median (range)	5 (0–18)	5 (0–19)	----	
Cut-off ≥8, %	26.5	26.6	----	
HADS – depression (scale: 0–21)	*N* = 131	*N* = 94	----	
Mean (SD)	3.7 (3.7)	4.1 (4.1)	7.37 (4.2)	*N* = 4516 [23]
			7.0 (2.9)	*N* = 269 [24]
Median (range)	2 (0–15)	3 (0–16)	----	
Cut-off ≥8, %	16.7	19.2	----	
CES-D (scale: 0–60)	*N* = 131	*N* = 95		
Mean (SD)	12.8 (9.6)	13.0 (11.6)	18.5 (10.1)	*N* = 51 [22]
			20 (12.1)	*N* = 463–472 [21]
Median (range)	10 (0–40)	9 (0–43)	----	
< 16, %	65.7	65.3	----	
16–21, %	16.8	12.6	----	
> 21, %	17.6	22.1	----	
FSMC (scale: 20–100)			----	
Cognitive	*N* = 66	*N* = 95	----	
Mean (SD)	23.0 (11.0)	22.2 (10.8)	----	
Median (range)	21.5 (10–50)	18 (10–50)	----	
Motor	*N* = 131	*N* = 98	----	
Mean (SD)	23.6 (11.1)	24.1 (11.8)	----	
Median (range)	22.9 (10–49)	20.5 (10–50)	----	
Total	*N* = 66	*N* = 95		
Mean (SD)	47.3 (21.9)	46.1 (21.9)	65.2 (17.5)	*N* = 51 [22]
Median (range)	47.5 (20–98)	39 (20–100)	----	
SDMT Total (scale: 0–110)	*N* = 116	*N* = 75		
Mean (SD)	47.9 (12.6)	51.5 (14.4)	49.06 (10.01)	*N* = 51 [22]
			51 (13.0)	*N* = 159 [26]
			47.66 (14.71)	*N* = 100 [25]
			55.3 (12.17)	*N* = 22 [27]
Median (range)	49 (16–76)	51 (15–95)	----	

*SD* standard deviation, *EDSS* expanded disability status scale, *FAMS* Functional Assessment of Multiple Sclerosis, *HADS* Hospital Anxiety and Depression Scale, *CES-D* Center for Epidemiologic Studies Depression Scale, *SDMT* The Symbol Digit Modalities Test, *FSMC* Fatigue Scale for Motor and Cognitive Functions

In general, functional health status appeared higher, whereas depression and fatigue scores were lower compared to other cohorts of MS patients [15, 19–25]. Cognitive function was comparable to prior studies (Table 4) [22, 25–27].

### Characterisation of patients with premature study discontinuation

Thirty-four patients (23.8%) prematurely discontinued the study. Nine patients were lost to follow-up, 6 patients discontinued due to adverse events (AEs), 4 withdrew their consent and 2 patients switched to another application method. For 13 patients, the reasons for discontinuation were specified by the attending physicians as follows: 6 patients switched to another medication, 2 patients stopped treatment with IFN beta-1b, 1 stopped using the BETACONNECT®, 2 were not-compliant, 1 patient got pregnant, and 1 discontinued at his/her own request. Table 5 illustrates baseline characteristics for patients prematurely discontinuing the study and those completing the study. Patients prematurely discontinuing were younger, had a shorter duration of disease, were more likely to be women, more likely to have been treated with IFNbeta-1b previously, and more likely to be participating in the BETAPLUS programme.

**Table 5:** Baseline characteristics among patients who prematurely discontinued and patients who completed the study

Characteristic	Patients with premature discontinuation (*N* = 34)	Patients completing the study (*N* = 109)
Age, years		
Mean (SD)	38.8 (10.7)	41.9 (11.6)
Median (range)	36 (21–64)	42 (21–79)
Gender, n (%)		
Women	26 (76.5)	73 (67.0)
Men	8 (22.5)	36 (33.0)
Duration of disease, months	*N* = 26	*N* = 76
Mean (SD)	44.8 (60.7)	61.6 (76.5)
Median (range)	24.8 (0.0–274.9)	33.6 (0.0–372.6)
EDSS, median (range))	*N* = 28	*N* = 96
	2.3 (0–6.0);	2.0 (0–6.5);
Previous treatment, n (%)		
Betaferon®	30 (88.2)	76 (69.7)
Other treatment	2 (5.9)	4 (3.7)
No previous treatment	2 (5.9)	29 (26.6)
Previous usage of auto-injector for Betaferon® treatment among patients who received Betaferon® previously, n (%)	*N* = 30	*N* = 76
Any	23 (76.7)	61 (80.3)
BETACOMFORT®	15 (60.0)	36 (59.0)
BETAJECT Comfort®	8 (32.0)	12 (19.7)
BETAJECT lite®	-	3 (4.9)
Other	-	10 (16.4)
BETAPLUS® participation, n (% of FAS)	26 (76.5)	61 (56.0)

*SD* standard deviation, *EDSS* expanded disability symptom scale, *FAS* full analysis set

Fourteen (41.2%) patients, who prematurely discontinued, still used the BETACONNECT® at their individual last visit before study discontinuation. Thirteen of the 18 patients (72.2%) who no longer used the BETACONNECT® also stopped IFNbeta-1b treatment.

### Adverse events

A total of 111 treatment-emergent adverse events (TEAEs) occurred in 51 patients (35.6% of 143 patients). TEAEs observed in >5% of all patients were erythema (20.3%) and influenza like illness (5.6%).

Additional information about further injection related data and use of additional electronic features of the BETACONNECT® are provided in the Additional file 7.

## Discussion

The BETAEVAL study investigated adherence of MS patients to IFN beta-1b treatment using the fully electronic BETACONNECT® autoinjector. Three quarters of patients still used the autoinjector at week 24. 57.9% of patients with at least one data readout from the device injected ≥80% of their doses. Among participitants with injection data at week 24, both the proportion of adherent patients (80.5%) and the mean compliance (defined as the mean percentage of injections administered: 92.9%) were high. Patients expressed a high level of satisfaction with the BETACONNECT®, more than 90% rated it as user-friendly and more than 80% preferred it over their previous way of injection.

Continuous treatment on a regular basis with injectable DMDs is essential to effectively control MS disease activity. However, treatment adherence remains a challenge and several population-based studies revealed that it is far from optimal. The “global adherence project”, a multinational, observational multicentre phase 4 study used a questionnaire-based approach to evaluate adherence to the commercially available DMDs [7]. Patients not missing a single DMD injection within 4 weeks before the study were considered adherent. Of the 2566 patients included in the analysis, 75% were adherent to their therapy [7]. A recent German retrospective cohort study, including data from 50,057 MS patients, showed that less than 40% of the patients took >80% of their prescribed medication over the observation period of 2 years [6]. Similarly, in a retrospective cohort study with 1606 patients from the US only 27–41% of patients in each year had a medication possession ratio of ≥85% and were thus considered adherent during the 3-year observation period [5]. Furthermore, a prospective study with 199 MS patients in Australia reported that 73% of patients missed doses during a mean follow-up period of 2.4 years [3].

Reasons causing non-adherence to injectable DMDs are often directly related to the drugs and their delivery systems [8]. Discomfort associated with treatment, such as pain at the injection site, local skin reactions, “flu-like” symptoms and fatigue as well as injection anxiety, may cause patients to intentionally skip doses or even discontinue treatment. Additionally, non-intentional forgetfulness or carelessness may contribute to non-adherence [5].

An important means of improving adherence is the development of injection devices aimed at increasing tolerability of the injections [10]. Currently, two fully electronic devices are available, one for the self-injection of IFN beta-1a (RebiSmart® [10]) and one for IFN beta-1b (BETACONNECT® [11]). Both devices share many features including e.g. variable injection speed and depths, reminder function, etc. The RebiSmart® has a display, appears somewhat bulkier and is heavier than the BETACONNECT®, which in contrast also offers the opportunity for data transfer into the myBETAapp®, allowing for personalised documentations of injection-related and wellness-related data. We may speculate, that depending on individual patients preferences and needs, these differences may influence injection behaviour and thus adherence. Patients with impaired vision for example might prefer a device without display and the slim shape of the BETACONNECT® might ease handling among patients with physical impairments of hands and/or arms.

Two observational studies with a retrospective design assessed adherence among RRMS patients by evaluating data from RebiSmart® that were returned for replacement [28, 29]. In both studies adherence was defined as the number of injections recorded by the device divided by the number of injections scheduled in the observation period (corresponding to our compliance definition). One was an audit of 225 patients in the UK and Ireland who had used the device for a minimum of 24 months. The mean age of the population was 44.1 years and 73% were women. At 24 months, 95.0% of the scheduled doses were injected. The proportion of patients that administered ≥80% of their doses was 91.1% [28]. The other study was conducted in Spain and included 258 patients with a mean age of 41 years, 68% were women. In this study, 92.6% of the scheduled doses were injected over a follow-up period of 3.1 years. 86.8% of the patients administered ≥80% of their prescribed doses [29].

Both studies are flawed by their retrospective design. The study from the UK and Ireland only enrolled patients who were still using the injection device after 2 years [28]. This study uses a “completers approach”, disregarding patients who prematurely stopped using the device and patients who simply did not return their devices. The study from Spain considered patients returning their devices for replacement as well as those prematurely terminating usage and returning their devices [29]. Hence, this study does not account for patients stopping usage early, refusing to return their devices or not consenting to study participation. Both approaches bias the results towards higher adherence compared to the whole cohort started on the device. The percentage adherence measure used in these studies is similar to the variable “compliance” in our study.

Prospective studies with the RebiSmart® covering shorter follow-up periods acknowledged patients prematurely stopping device usage. One multicentre, observational phase IV study with 119 patients in the intention-to treat (ITT) population conducted in Italy (BRIDGE study) assessed patient adherence to the device over 12 weeks [30]. All patients were diagnosed with RRMS and had received prior treatment with injectable DMDs. The mean age was 37.9 years and 75.6% were women. At week 12, 88.2% of the patients administered ≥80% of the scheduled injections over the course of the study, as recorded by the autoinjector. Long-term-adherence among 57 of these patients was assessed in the RIVER study [31], a real life-extension study of the BRIDGE study. Overall adherence during the mean observation period of 20.5 ± 5.7 months was 79.8% [31]. The RIVER study is an example underlining the difficulty in retaining patients in real-life studies over extended periods of time.

The MEASURE study, a Canadian multicentre, observational, phase IV study, enrolled 162 patients with RRMS using the RebiSmart® for a maximum of 96 weeks. Patients were only eligible if they had not received any previous DMD treatment. Adherence was defined as the administration of ≥80% of expected injections over the entire study period as recorded by the autoinjector. Compliance was defined as the percentage of administered injections during the actual treatment period until treatment discontinuation. The mean age of the study population was 37.4 years, 75.3% were women and the mean time since MS diagnosis was 24 months. In the modified ITT population (*n* = 158), 91.8 and 82.9% of patients were adherent at weeks 12 and 24, respectively [32]. At week 24, 13.9% of participants had discontinued treatment. Among patients remaining on treatment the proportion of participants with ≥80% compliance remained high throughout the study (week 12: 95.6%, week 24: 92.4%). First data from week 96 was presented recently. At week 96, adherence had dropped to 69.5%, whereas the proportion of patients with ≥80% compliance was still high (85.5%) [26].

Data on adherence to treatment with RebiSmart® in Germany are available from a prospective, non-interventional, multicentre READOUTsmart study with 368 patients included in the analysis. In this study, quantitative adherence was defined as the proportion of scheduled injections that were actually administered, as documented by the RebiSmart®. Study participants had a mean age of 36.8 years, 69.6% were women and the mean time since first diagnosis of MS was 2.7 years. Quantitative adherence was 85.3% for the entire study duration of 24 months. A quantitative adherence ≥85% was reported for 72.0 and 65.5% at month 12 and month 24, respectively [33].

In the BETAEVAL study more patients had prematurely terminated study participation than in the MEASURE study at 24 weeks [32] (23.4% vs. 13.9%). This may be due to differences among study participants with patients in our study being slightly older (mean age 41.2 vs. 37.4 years), having a longer mean disease duration (57.5 vs. 24.0 months), and being mostly pretreated with DMDs (74.1% previously treated with IFN beta-1b vs. 100% treatment naïve) compared to the participants in the MEASURE study [32]. Furthermore, pretreatment was also very different in both the German READOUTsmart study, with only 21.5% of patients having been on DMDs previously [33] and in the BRIDGE study with almost two thirds of patients switching from intramuscular to subcutaneous injection [30]. In addition, follow-up periods in the READOUTsmart [33] and the BRIDGE study [30] differed from ours, precluding a direct comparison between the adherence measures.

Patients with a longer disease duration and treatment history with injectable DMDs may be more impatient and have higher expectations due to their previous experiences compared to DMD-naïve patients, resulting in a higher proportion of patients prematurely terminating the BETAEVAL study. On the other hand, “first-time users” of DMDs may be more motivated and determined to stick to the treatment regimen in order to change their natural disease course. This is corroborated by results from our stratified analysis, indicating a higher proportion of patients prematurely terminating the study among experienced patients compared to naïve patients (Fig. 2). In addition, we identified “no previous treatment” as an important predictor of both persistence and adherence by applying post-hoc prediction models.

Another potential factor influencing treatment discontinuation in our study might be the availability of new oral drugs. Patients in the BRIDGE study were recruited in 2009/2010, whereas our study started in 2014. In this time period three new oral drugs for MS treatment were approved in Europe: fingolimod (2011), teriflunomide (2013) and dimethyl fumarate (2014), possibly increasing the incentive for patients to switch to oral drugs.

The proportion of adherent patients and the mean compliance among patients in the BETAEVAL study using the BETACONNECT® were high, indicating that the device is a useful and well-received tool to support patients with their injections. The BETACONNECT® was designed to further improve patients’ injection experience and to overcome some of the barriers leading to non-adherence such as needle phobia or injection anxiety. In fact, patients in the BETAEVAL study indicated a high degree of satisfaction with the BETACONNECT®. There was no difference in the perception of injection-related pain between the BETACONNECT® and the previous way of injection; however, only 9.1% of patients with corresponding questionnaire used analgesics prior to their injection at week 24, whereas 14.6% indicate this before the study start and 17.2% at week 4.

Patients rated the BETACONNECT® as user-friendly and at the initial visit, a majority of patients already indicated that they felt confident with the BETACONNECT® compared with their previous way of injection and that they preferred it to their previous way. The proportion of participants “strongly agreeing” to the latter statements increased over time, indicating that their initial impression was confirmed and that their appreciation grew while further familiarizing themselves with the device. The results concerning satisfaction are in line with a survey-based study conducted on 118 patients using the BETACONNECT® in Germany [11]. Among those 92% indicated that they were very confident or confident using the autoinjector and almost half of them stated, that the ease-of-use was the primary reason for their satisfaction with the device. High levels of satisfaction with the BETACONNECT® may be an important factor contributing to the high adherence and compliance seen in our study.

The strengths of the BETAEVAL study include the prospective data collection, the large study population, and the observational study design enrolling patients representative of German MS patients thus allowing a real-world picture of the treatment situation in Germany to be drawn. Furthermore, key characteristics and results from questionnaires suggest that the participants in the BETAEVAL study are comparable to other cohorts of patients with relapsing forms of MS with a slightly higher functional health status and a slightly lower level of depression and anxiety [15, 19–27].

However, some limitations need to be considered when interpreting the results. First, the follow-up of 6 months was rather short. However, this study was designed to allow for an early evaluation of the BETACONNECT® and its usage in a real-world setting, in order to be able to address potential problems early. In addition, a follow up study is currently ongoing to evaluate adherence among MS patients using the device for injection of IFN beta-1b over up to 2 years [34]. Second, injection data from the BETACONNECT® were not available for all study participants and not at all follow-up visits. Finally, the BETAEVAL study lacks a control group, precluding comparison of adherence between patients using the BETACONNECT® and a different way of injecting IFN beta-1b; however, this was not the aim of the study.

## Conclusions

The majority of study participants used the fully electronic BETACONNECT® autoinjector throughout the study. Adherence, persistence and compliance were high. Most participants were very satisfied with the device, the vast majority also giving high ratings for user friendliness, feeling confident in using it and preferring it over their previously used device. Hence, the BETACONNECT may be a useful tool to support patients in following their treatment regimen with IFN beta-1b.

## Additional files

Additional file 1: Patient questionnaire regarding secondary outcomes. Description of data: questions regarding satisfaction, user friendliness, injection site related pain as well as preference of and confidence in using the BETACONNECT®. (PDF 254 kb)

Additional file 2: Adherence, satisfaction and functional health status among patients with multiple sclerosis using the BETACONNECT® autoinjector: a prospective observational cohort study. Description of data: description of the rating scales used for documentation of other patient-reported outcomes. (DOCX 17 kb)

Additional file 3: Table S1. Satisfaction with the BETACONNECT® among participants in the BETAEVAL study - stratified analyses. Description of data: data on analyses stratified by age, gender, EDSS baseline score, previous treatment with INF beta-1b, and BETAPLUS participation. (DOCX 21 kb)

Additional file 4: Table S2. Injection related pain – stratified analyses. Description of data: data on analyses stratified by age, gender, EDSS baseline score, previous treatment with INF beta-1b, and BETAPLUS participation. (DOCX 18 kb)

Additional file 5: Table S3. Prophylactic use of analgesics prior to injection – stratified analyses. Description of data: data on analyses stratified by age, gender, EDSS baseline score, previous treatment with INF beta-1b, and BETAPLUS participation. (DOCX 16 kb)

Additional file 6: Table S4. Patient-related outcome measures – stratified analyses. Description of data: data on analyses stratified by age, gender, EDSS baseline score, previous treatment with INF beta-1b, and BETAPLUS participation. (DOCX 36 kb)

Additional file 7: Adherence, satisfaction and functional health status among patients with multiple sclerosis using the BETACONNECT® autoinjector: a prospective observational cohort study. Description of data: results on further injection related data and use of electronic features of the BETACONNECT® are provided. (DOCX 12 kb)

## Abbreviations

AE: Adverse event
CES-D: Centre for Epidemiologic Studies Depression Scale
CI: Confidence interval
CIS: Clinically isolated syndrome
DMD: Disease modifying drug
eCRFs: Electronic case report forms
EDC: Electronic data capture system
EDSS: Expanded disability status scale
FAMS: Functional assessment of multiple sclerosis
FAS: Full analysis set
FSMC: Fatigue Scale for Motor and Cognitive Functions
HADS: Hospital Anxiety and Depression Scale
IFN: Interferon
im: Intramuscular
ITT: Intention-to-treat
MS: Multiple sclerosis
RRMS: Relapsing remitting multiple sclerosis
SAF: Safety analysis set
sc: Subcutaneous
SD: Standard deviation
SDMT: Symbol Digit Modalities Test
TEAE: Treatment-emergent adverse events
