# Supplementary material for: Adherence, satisfaction and functional health status among patients with multiple sclerosis using the BETACONNECT® autoinjector: a prospective observational cohort study
Source: BMC Neurol. 2017 Sep 6;17:174. doi: 10.1186/s12883-017-0953-8 (PMC5588619; doi:10.1186/s12883-017-0953-8)
Supplement: Supplementary file 1 — Patient questionnaire regarding secondary outcomes. Description of data: questions regarding satisfaction, user friendliness, injection site related pain as well as preference of and confidence in using the BETACONNECT®. (PDF 254 kb) [file 12883_2017_953_MOESM1_ESM.pdf]

Patient questionnaire regarding secondary outcomes:

PART A: Prior to BETACONNECT® training (only initial visit):

1. Overall how would you rate your satisfaction with your current way of Betaferon® injection on a scale from 0 to 10, where “0” means “not satisfied at all” and “10” means “entirely satisfied”?

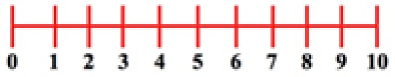

2. Overall I consider my current way of Betaferon® injection user-friendly:
    a. Strongly agree
    b. Agree
    c. Neutral
    d. Disagree
    e. Strongly disagree

3. With your current way of Betaferon® injection are you using any analgesics prior to injection?
    a. No
    b. Yes

4. Overall how would you rate your current intensity of injection site-related pain on a scale from 0 to 10, where “0” means “no pain at all” and “10” means the “worst possible pain”?

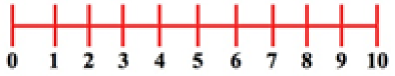

PART B: Directly after the BETACONNECT® training during initial visit

1. Overall I <u>prefer</u> the BETACONNECT® auto-injector device over my previous way of Betaferon® injection:
    a. Strongly agree
    b. Agree
    c. Neutral
    d. Disagree
    e. Strongly disagree

2. Overall I feel <u>confident</u> in using the BETACONNECT® for delivering Betaferon® compared to my previous way of Betaferon® injection:
    a. Strongly agree
    b. Agree
    c. Neutral
    d. Disagree
    e. Strongly disagree

3. Overall how would you rate your <u>satisfaction</u> with the BETACONNECT® auto-injector device on a scale from 0 to 10, where “0” means “not satisfied at all” and “10” means “entirely satisfied”?

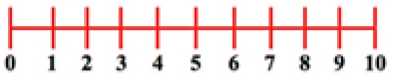

PART B: at Follow up visits and final visit

1. Overall I prefer the BETACONNECT® auto-injector device over my previous way of Betaferon® injection:
    a. Strongly agree
    b. Agree
    c. Neutral
    d. Disagree
    e. Strongly disagree

2. Overall I feel confident in using the BETACONNECT® for delivering Betaferon® compared to my previous way of Betaferon® injection:
    a. Strongly agree
    b. Agree
    c. Neutral
    d. Disagree
    e. Strongly disagree

3. Overall how would you rate your satisfaction with the BETACONNECT® auto-injector device on a scale from 0 to 10, where “0” means “not satisfied at all” and “10” means “entirely satisfied”?

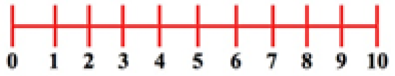

4. Overall the BETACONNECT® auto-injector device is user-friendly:
    a. Strongly agree
    b. Agree
    c. Neutral
    d. Disagree
    e. Strongly disagree

5. When using the BETACONNECT® are you using any analgesics prior to injection?
    a. No
    b. Yes

6. Overall, when using the BETACONNECT® auto-injector, how would you rate your intensity of injection site related pain on a scale from 0 to 10, where “0” means “no pain at all” and “10” means the “worst possible pain”?

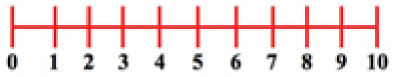

7. Are you using the electronic features of the BETACONNECT®?
    a. No
    b. Yes, If yes, please specify …
