# Supplementary material for: Adherence, satisfaction and functional health status among patients with multiple sclerosis using the BETACONNECT® autoinjector: a prospective observational cohort study
Source: BMC Neurol. 2017 Sep 6;17:174. doi: 10.1186/s12883-017-0953-8 (PMC5588619; doi:10.1186/s12883-017-0953-8)
Supplement: Supplementary file 2 — Adherence, satisfaction and functional health status among patients with multiple sclerosis using the BETACONNECT® autoinjector: a prospective observational cohort study. Description of data: description of the rating scales used for documentation of other patient-reported outcomes. (DOCX 17 kb) [file 12883_2017_953_MOESM2_ESM.docx]

**Supplementary methods to:**

*Kleiter I., et al.: Adherence, satisfaction and functional health status among patients with multiple sclerosis using the BETACONNECT^®^ autoinjector: a prospective observational cohort study*

**The following rating scales were used for documentation of other patient-reported outcomes:**

*FAMS:* the FAMS Version 4 quality of life questionnaire consists of 58 items grouped into the domains mobility, symptoms, emotional well-being, general contentment, thinking and fatigue, family/social well-being, and additional concerns. Each item is scored on a five-point scale ranging from 0 to 4, and the questions cover the patient´s experience over the previous 7 days [1].

*HADS:* The HADS is a fourteen-item scale with seven items relating to anxiety and seven relating to depression. Each item on the questionnaire is scored from 0-3; hence a person can score between 0 and 21 for either anxiety or depression. The cut-off point for depression or anxiety is 8/21 [2].

*CES-D:* The CES-D measures symptoms of depression experienced during the past week. It includes 20 items comprising six scales: depressed mood, feelings of guilt and worthlessness, feelings of helplessness and hopelessness, psychomotor retardation, loss of appetite, and sleep disturbance. Each item can be scored from 0-3 allowing for a possible range of scores from 0 to 60, with the scores indicating the presence of more symptomatology. A score above 16 suggests a mild to moderate level of depressive symptoms, a score above 21 suggests major depressive symptoms [3].

*FSMC:* the FSMC was designed to evaluate cognitive and physical fatigue among MS patients. The questionnaire consists of 20 items, categorized into two subscales for cognitive and physical fatigue. Each item is scored on a 5-point Likert scale. This allows for a score range from 20 to 100 for the combined scale (10 to 50 for each subscale). The cut-off values for the FSMC are as follows: sum score (≥43 mild fatigue, ≥53 moderate fatigue, ≥63 severe fatigue), cognitive score (≥22 mild cognitive fatigue, ≥28 moderate cognitive fatigue, ≥34 severe cognitive fatigue), physical fatigue (≥22 mild physical fatigue, ≥27 moderate physical fatigue, ≥32 severe physical fatigue) [4].

**References:**

1. Cella DF, Dineen K, Arnason B, Reder A, Webster KA, karabatsos G, Chang C, Lloyd S, Steward J, Stefoski D: **Validation of the functional assessment of multiple sclerosis quality of life instrument**. *Neurology* 1996, **47**(1):129-139.

2. Zigmond AS, Snaith RP: **The hospital anxiety and depression scale**. *Acta Psychiatr Scand* 1983, **67**(6):361-370.

3. Radloff L: **The CES-D scale: a self-report depressive scale for research in the general population.** *J Appl Psychol Measurement* 1977, **1**:385-401.

4. Penner IK, Raselli C, Stocklin M, Opwis K, Kappos L, Calabrese P: **The Fatigue Scale for Motor and Cognitive Functions (FSMC): validation of a new instrument to assess multiple sclerosis-related fatigue**. *Mult Scler* 2009, **15**(12):1509-1517.
