# Supplementary material for: Adherence, satisfaction and functional health status among patients with multiple sclerosis using the BETACONNECT® autoinjector: a prospective observational cohort study
Source: BMC Neurol. 2017 Sep 6;17:174. doi: 10.1186/s12883-017-0953-8 (PMC5588619; doi:10.1186/s12883-017-0953-8)
Supplement: Supplementary file 3 — Satisfaction with the BETACONNECT® among participants in the BETAEVAL study - stratified analyses. Description of data: data on analyses stratified by age, gender, EDSS baseline score, previous treatment with INF beta-1b, and BETAPLUS participation. (DOCX 21 kb) [file 12883_2017_953_MOESM3_ESM.docx]

**Supplementary Table 1:** Satisfaction with the BETACONNECT^®^ among participants in the BETAEVAL study - stratified analyses

| **Satisfaction with…,** | **…way of injection before study start** | | **…BETACONNECT^®^** | | | | | |
| --- | --- | --- | --- | --- | --- | --- | --- | --- |
|  | **Initial visit** | | **Follow-up visit after 4 weeks** | | **Follow-up visit after 12 weeks** | | **Follow-up visit after 24 weeks** | |
|  | n | NAS range 0-10 | n | NAS range 0-10 | n | NAS range 0-10 | n | NAS range 0-10 |
| **Age** |  |  |  |  |  |  |  |  |
| < 40 Mean (SD) | 39 | 7.3 (1.8) | 53 | 8.3 (2.0) | 48 | 8.2 (1.5) | 43 | 8.2 (1.5) |
| ≥ 40 Mean (SD) | 54 | 7.4 (2.2) | 62 | 8.4 (1.7) | 61 | 8.7 (1.5) | 56 | 8.6 (1.4) |
| < 40 Median (range) | 39 | 8 (1, 10) | 53 | 9 (1, 10) | 48 | 8 (3, 10) | 43 | 9 (3, 10) |
| ≥ 40 Median (range) | 54 | 8 (1, 10) | 62 | 9 (3, 10) | 61 | 9 (2, 10) | 56 | 9 (3, 10) |
| **Gender** |  |  |  |  |  |  |  |  |
| Female Mean (SD) | 62 | 7.4 (2.1) | 80 | 8.3 (1.8) | 74 | 8.4 (1.4) | 67 | 8.5 (1.3) |
| Male Mean (SD) | 31 | 7.5 (1.9) | 35 | 8.4 (2.0) | 35 | 8.5 (1.7) | 32 | 8.3 (1.7) |
| Female Median (range) | 62 | 8 (1, 10) | 80 | 9 (1, 10) | 74 | 9 (2, 10) | 67 | 9 (3, 10) |
| Male Median (range) | 31 | 8 (1, 10) | 35 | 9 (3, 10) | 35 | 9 (3, 10) | 32 | 9 (3, 10) |
| **EDSS baseline score** |  |  |  |  |  |  |  |  |
| < 3 Mean (SD) | 65 | 7.3 (1.8) | 74 | 8.2 (2.0) | 73 | 8.3 (1.6) | 64 | 8.4 (1.5) |
| ≥ 3 Mean (SD) | 22 | 7.6 (2.5) | 28 | 8.3 (1.8) | 26 | 9.0 (1.2) | 24 | 8.3 (1.4) |
| < 3 Median (range) | 65 | 8 (1, 10) | 74 | 9 (1, 10) | 73 | 9 (2, 10) | 64 | 9 (3, 10) |
| ≥ 3 Median (range) | 22 | 8.5 (1, 10) | 28 | 8 (3, 10) | 26 | 9 (6, 10) | 24 | 8 (5, 10) |
| **Previous treatment with IFN beta-1b** |  |  |  |  |  |  |  |  |
| Yes Mean (SD) | 93 | 7.4 (2.0) | 85 | 8.3 (1.9) | 77 | 8.3 (1.6) | 69 | 8.4 (1.5) |
| No Mean (SD) | NA | NA | 30 | 8.5 (1.9) | 32 | 8.8 (1.1) | 30 | 8.6 (1.4) |
| Yes Median (range) | 93 | 8 (1, 10) | 85 | 9 (1, 10) | 77 | 9 (2, 10) | 69 | 9 (3, 10) |
| No Median (range) | NA | NA | 30 | 9 (3, 10) | 32 | 9 (7, 10) | 30 | 9 (5, 10) |
| **BETAPLUS participation** |  |  |  |  |  |  |  |  |
| Yes Mean (SD) | 58 | 7.4 (2.2) | 65 | 8.5 (1.7) | 61 | 8.4 (1.5) | 56 | 8.5 (1.4) |
| No Mean (SD) | 35 | 7.3 (1.7) | 50 | 8.1 (2.0) | 48 | 8.5 (1.5) | 43 | 8.3 (1.5) |
| Yes Median (range) | 58 | 8 (1, 10) | 65 | 9 (3, 10) | 61 | 9 (2, 10) | 56 | 9 (3, 10) |
| No Median (range) | 35 | 7 (3, 10) | 50 | 9 (1, 10) | 48 | 9 (3, 10) | 43 | 8 (3, 10) |

*NAS* numerical analogue scale, *SD* standard deviation, *IFN* interferon
