# Supplementary material for: Adherence, satisfaction and functional health status among patients with multiple sclerosis using the BETACONNECT® autoinjector: a prospective observational cohort study
Source: BMC Neurol. 2017 Sep 6;17:174. doi: 10.1186/s12883-017-0953-8 (PMC5588619; doi:10.1186/s12883-017-0953-8)
Supplement: Supplementary file 4 — Injection related pain – stratified analyses. Description of data: data on analyses stratified by age, gender, EDSS baseline score, previous treatment with INF beta-1b, and BETAPLUS participation. (DOCX 18 kb) [file 12883_2017_953_MOESM4_ESM.docx]

**Supplementary Table 2:** Injection related pain – stratified analyses

| **Injection related pain with…,** | **…. previous way of injection** | | **…BETACONNECT®** | | | | | |
| --- | --- | --- | --- | --- | --- | --- | --- | --- |
|  | **Initial visit** | | **Follow-up visit after 4 weeks** | | **Follow-up visit after 12 weeks** | | **Follow-up visit after 24 weeks** | |
| **Age** | n |  | n |  | n |  | n |  |
| < 40 Mean (SD) | 40 | 4.0 (2.4) | 53 | 4.0 (2.3) | 50 | 4.3 (2.4) | 43 | 4.6 (2.6) |
| ≥ 40 Mean (SD) | 54 | 4.3 (2.7) | 61 | 3.2 (2.6) | 60 | 3.8 (2.8) | 55 | 3.7 (2.6) |
| < 40 Median (range) | 40 | 3 (0, 9) | 53 | 4 (0, 8) | 50 | 5 (0, 9) | 43 | 5 (0, 9.5) |
| ≥ 40 Median (range) | 54 | 4 (0, 10) | 61 | 3 (0, 10) | 60 | 3 (0, 10) | 55 | 3 (0, 10) |
| **Gender** |  |  |  |  |  |  |  |  |
| Female Mean (SD) | 63 | 4.5 (2.6) | 79 | 4.0 (2.5) | 74 | 4.5 (2.7) | 66 | 4.5 (2.8) |
| Male Mean (SD) | 31 | 3.6 (2.5) | 35 | 2.7 (2.2) | 36 | 3.1 (2.1) | 32 | 3.2 (2.1) |
| Female Median (range) | 63 | 5 (0, 10) | 79 | 4 (0, 10) | 74 | 5 (0, 10) | 66 | 4 (0, 10) |
| Male Median (range) | 31 | 3 (0, 9) | 35 | 2 (0, 8) | 36 | 3 (0, 9) | 32 | 3 (0, 8) |
| **EDSS baseline score** |  |  |  |  |  |  |  |  |
| < 3 Mean (SD) | 66 | 4.0 (2.4) | 74 | 3.7 (2.5) | 74 | 3.8 (2.5) | 64 | 4.2 (2.7) |
| ≥ 3 Mean (SD) | 22 | 4.8 (2.8) | 27 | 2.8 (2.3) | 26 | 4.4 (3.1) | 23 | 4.0 (3.0) |
| < 3 Median (range) | 66 | 4 (0, 10) | 74 | 3 (0, 10) | 74 | 3 (0, 9) | 64 | 4 (0, 10) |
| ≥ 3 Median (range) | 22 | 5 (0,10) | 27 | 3 (0, 8) | 26 | 5 (0, 10) | 23 | 4 (0, 9) |
| **Previous treatment with IFN-beta 1b** |  |  |  |  |  |  |  |  |
| Yes Mean (SD) | 94 | 4.2 (2.5) | 85 | 3.5 (2.5) | 78 | 3.6 (2.5) | 69 | 3.9 (2.7) |
| No Mean (SD) | NA | NA | 29 | 4.0 (2.2) | 32 | 5.0 (2.6) | 29 | 4.7 (2.6) |
| Yes Median (range) | 94 | 4 (0, 10) | 85 | 3 (0, 10) | 78 | 3 (0, 9) | 69 | 3 (0, 10) |
| No Median (range) | NA | NA | 29 | 4 (0, 8) | 32 | 5 (0, 10) | 29 | 5 (0, 9) |
| **BETAPLUS participation** |  |  |  |  |  |  |  |  |
| Yes Mean (SD) | 58 | 4.3 (2.7) | 64 | 4.0 (2.4) | 62 | 4.5 (2.6) | 55 | 4.3 (2.6) |
| No Mean (SD) | 36 | 3.9 (2.3) | 50 | 3.2 (2.5) | 48 | 3.5 (2.5) | 43 | 3.8 (2.7) |
| Yes Median (range) | 58 | 4 (0, 10) | 64 | 4 (0, 10) | 62 | 5 (0, 10) | 55 | 4 (0, 9) |
| No Median (range) | 36 | 3.5 (0, 9) | 50 | 3 (0, 8) | 48 | 3 (0, 9) | 43 | 3 (0, 10) |

*NAS* numerical analogue scale, *SD* standard deviation, *IFN* interferon
