# Supplementary material for: Adherence, satisfaction and functional health status among patients with multiple sclerosis using the BETACONNECT® autoinjector: a prospective observational cohort study
Source: BMC Neurol. 2017 Sep 6;17:174. doi: 10.1186/s12883-017-0953-8 (PMC5588619; doi:10.1186/s12883-017-0953-8)
Supplement: Supplementary file 5 — Prophylactic use of analgesics prior to injection – stratified analyses. Description of data: data on analyses stratified by age, gender, EDSS baseline score, previous treatment with INF beta-1b, and BETAPLUS participation. (DOCX 16 kb) [file 12883_2017_953_MOESM5_ESM.docx]

**Supplementary Table 3:** Prophylactic use of analgesics prior to injection – stratified analyses

| **Prophylactic use of analgesics prior to injection with…,** | **… previous way of injection** | | **… BETACONNECT®** | | | | | |
| --- | --- | --- | --- | --- | --- | --- | --- | --- |
|  | **Initial visit**  (Patients with Questionnaire A) | | **4 weeks**  (Patients with Questionnaire B) | | **12 weeks**  (Patients with Questionnaire B) | | **24 weeks**  (Patients with Questionnaire B) | |
|  | n | % | n | % | n | % | n | % |
| **Age** |  |  |  |  |  |  |  |  |
| < 40 | 41 | 9.8 | 54 | 11.1 | 50 | 20.0 | 43 | 16.3 |
| ≥ 40 | 55 | 18.2 | 62 | 22.6 | 61 | 14.8 | 56 | 3.6 |
| **Gender** |  |  |  |  |  |  |  |  |
| Female | 65 | 18.5 | 81 | 19.8 | 75 | 21.3 | 67 | 11.9 |
| Male | 31 | 6.5 | 35 | 11.4 | 36 | 8.3 | 32 | 3.1 |
| **EDSS baseline score** |  |  |  |  |  |  |  |  |
| < 3 | 67 | 14.9 | 74 | 14.9 | 74 | 13.5 | 64 | 9.4 |
| ≥ 3 | 23 | 17.4 | 28 | 21.4 | 26 | 19.2 | 24 | 12.5 |
| **Previous treatment with IFN beta-1b** |  |  |  |  |  |  |  |  |
| Yes | 96 | 14.6 | 86 | 17.4 | 78 | 10.3 | 69 | 5.8 |
| No | NA | NA | 30 | 16.7 | 33 | 33.3 | 30 | 16.7 |
| **BETAPLUS participation** |  |  |  |  |  |  |  |  |
| Yes | 58 | 17.2 | 66 | 22.7 | 63 | 19.1 | 56 | 8.9 |
| No | 38 | 10.5 | 50 | 10.0 | 48 | 14.6 | 43 | 9.3 |

*EDSS* expanded disability status scale, *IFN* interferon
