# Supplementary material for: Adherence, satisfaction and functional health status among patients with multiple sclerosis using the BETACONNECT® autoinjector: a prospective observational cohort study
Source: BMC Neurol. 2017 Sep 6;17:174. doi: 10.1186/s12883-017-0953-8 (PMC5588619; doi:10.1186/s12883-017-0953-8)
Supplement: Supplementary file 6 — Patient-related outcome measures – stratified analyses. Description of data: data on analyses stratified by age, gender, EDSS baseline score, previous treatment with INF beta-1b, and BETAPLUS participation. (DOCX 36 kb) [file 12883_2017_953_MOESM6_ESM.docx]

***Supplementary Table 4:*** Patient-related outcome measures – stratified analyses

| **Questionnaire** | | **Initial visit** | | **24-week visit** | |
| --- | --- | --- | --- | --- | --- |
|  | | n | Mean (SD) | n | Mean (SD) |
| **FAMS (scale: 0-176)**  Total score (without regard to additional concerns) | | | | | |
|  | **Total** | 192 | 135.5 (29.2) | 97 | 134.6 (33.7) |
|  | **Age** |  |  |  |  |
|  | < 40 | 62 | 143.68 (22.2) | 43 | 137.8 (28.8) |
|  | ≥ 40 | 67 | 127.9 (32.8) | 54 | 132.0 (37.3) |
|  | **Gender** |  |  |  |  |
|  | Female | 90 | 133.9 (28.7) | 66 | 132.2 (33.1) |
|  | Male | 39 | 139.2 (30.4) | 31 | 139.6 (35.0) |
|  | **EDSS baseline score** |  |  |  |  |
|  | < 3 | 79 | 146.6 (23.7) | 62 | 143.5 (27.4) |
|  | ≥ 3 | 31 | 110.3 (30.4) | 23 | 107.1 (36.0) |
|  | **Previous treatment with INF-beta 1b** |  |  |  |  |
|  | Yes | 93 | 136.1 (28.5) | 66 | 138.8 (30.8) |
|  | No | 36 | 134.0 (31.3) | 31 | 125.5 (38.3) |
|  | **Participation in BETAPLUS program** |  |  |  |  |
|  | Yes | 78 | 132.4 (31.7) | 55 | 127.8 (34.7) |
|  | No | 51 | 140.2 (24.4) | 42 | 143.4 (30.6) |
| **HADS – anxiety (scale: 0-21)** | | | | | |
|  | **Total** | 131 | 5.3 (4.1) | 94 | 5.4 (4.3) |
|  | **Age** |  |  |  |  |
|  | < 40 | 62 | 4.7 (3.6) | 43 | 5.3 (3.8) |
|  | ≥ 40 | 69 | 5.9 (4.4) | 51 | 5.5 (4.7) |
|  | **Gender** |  |  |  |  |
|  | Female | 91 | 5.6 (4.1) | 64 | 5.7 (4.6) |
|  | Male | 40 | 4.8 (4.0) | 30 | 4.7 (3.5) |
|  | **EDSS baseline score** |  |  |  |  |
|  | < 3 | 82 | 4.7 (3.9) | 58 | 4.6 (4.2) |
|  | ≥ 3 | 31 | 7.1 (3.9) | 24 | 6.5 (3.5) |
|  | **Previous treatment with INF beta-1b** |  |  |  |  |
|  | Yes | 96 | 5.2 (4.0) | 64 | 5.2 (4.1) |
|  | No | 35 | 5.6 (4.3) | 30 | 5.7 (4.6) |
|  | **Participation in BETAPLUS program** |  |  |  |  |
|  | Yes | 79 | 5.9 (4.2) | 52 | 6.5 (4.7) |
|  | No | 52 | 4.5 (3.7) | 42 | 4.0 (3.3) |
| **HADS – depression (scale: 0-21**) | | | | | |
|  | **Total** | 131 | 3.7 (3.7) | 94 | 4.1 (4.1) |
|  | **Age** |  |  |  |  |
|  | < 40 | 62 | 3.3 (3.4) | 43 | 3.7 (3.9) |
|  | ≥ 40 | 69 | 4.0 (3.9) | 51 | 4.4 (4.4) |
|  | **Gender** |  |  |  |  |
|  | Female | 91 | 3.8 (3.9) | 64 | 4.3 (4.1) |
|  | Male | 40 | 3.6 (3.2) | 30 | 3.7 (4.3) |
|  | **EDSS baseline score** |  |  |  |  |
|  | < 3 | 82 | 3.1 (3.7) | 58 | 3.2 (3.8) |
|  | ≥ 3 | 31 | 5.5 (3.6) | 24 | 6.5 (4.1) |
|  | **Previous treatment with INF beta-1b** |  |  |  |  |
|  | Yes | 96 | 3.7 (3.8) | 64 | 4.0 (4.3) |
|  | No | 35 | 3.6 (3.3) | 30 | 4.3 (3.8) |
|  | **Participation in BETAPLUS program** |  |  |  |  |
|  | Yes | 79 | 3.9 (3.9) | 52 | 4.8 (4.3) |
|  | No | 52 | 3.3 (3.3) | 42 | 3.2 (3.8) |
| **CES-D (scale: 0-60)** | | | | | |
|  | **Total** | 131 | 12.8 (9.6) | 95 | 13.0 (11.6) |
|  | **Age** |  |  |  |  |
|  | < 40 | 61 | 10.8 (8.6) | 43 | 12.0 (9.7) |
|  | ≥ 40 | 70 | 14.5 (10.1) | 52 | 13.8 (12.9) |
|  | **Gender** |  |  |  |  |
|  | Female | 92 | 13.0 (9.7) | 64 | 13.7 (12.2) |
|  | Male | 39 | 12.3 (9.4) | 31 | 11.5 (10.2) |
|  | **EDSS baseline score** |  |  |  |  |
|  | < 3 | 81 | 10.4 (8.6) | 59 | 10.8 (10.8) |
|  | ≥ 3 | 32 | 19.4 (9.5) | 24 | 19.5 (11.6) |
|  | **Previous treatment with INF beta-1b** |  |  |  |  |
|  | Yes | 96 | 12.6 (9.7) | 65 | 12.2 (11.3) |
|  | No | 35 | 13.5 (9.6) | 30 | 14.6 (12.2) |
|  | **Participation in BETAPLUS program** |  |  |  |  |
|  | Yes | 79 | 14.2 (9.9) | 53 | 15.6 (12.6) |
|  | No | 52 | 10.6 (8.7) | 42 | 9.7 (9.2) |
| **FSMC (scale: 20-100) – cognitive fatigue** | | | | | |
|  | **Total** | 66 | 23.0 (11.0) | 95 | 22.2 (10.8) |
|  | **Age** |  |  |  |  |
|  | < 40 | 31 | 18.2 (8.1) | 44 | 20.3 (9.0) |
|  | ≥ 40 | 35 | 27.2 (11.6) | 51 | 23.8 (12.0) |
|  | **Gender** |  |  |  |  |
|  | Female | 46 | 23.5 (11.4) | 65 | 23.5 (11.3) |
|  | Male | 20 | 21.6 (10.1) | 30 | 19.4 (9.2) |
|  | **EDSS baseline score** |  |  |  |  |
|  | < 3 | 35 | 18.7 (9.0) | 61 | 19.2 (9.2) |
|  | ≥ 3 | 19 | 31.4 (11.1) | 23 | 30.2 (11.1) |
|  | **Previous treatment with INF beta-1b** |  |  |  |  |
|  | Yes | 44 | 22.9 (11.5) | 65 | 21.2 (10.2) |
|  | No | 22 | 23.1 (10.3) | 30 | 24.2 (11.9) |
|  | **Participation in BETAPLUS program** |  |  |  |  |
|  | Yes | 44 | 25.5 (11.7) | 53 | 24.6 (11.2) |
|  | No | 22 | 17.9 (7.5) | 42 | 19.1 (9.5) |
| **FSMC (scale: 20-100) – motor fatigue** | | | | | |
|  | **Total** | 131 | 23.6 (11.1) | 98 | 24.1 (11.8) |
|  | **Age** |  |  |  |  |
|  | < 40 | 62 | 20.3 (9.8) | 44 | 21.0 (10.0) |
|  | ≥ 40 | 69 | 26.7 (11.4) | 54 | 26.6 (12.6) |
|  | **Gender** |  |  |  |  |
|  | Female | 91 | 25.0 (10.1) | 66 | 24.9 (11.8) |
|  | Male | 40 | 20.6 (10.8) | 32 | 22.5 (11.7) |
|  | **EDSS baseline score** |  |  |  |  |
|  | < 3 | 82 | 20.3 (9.7) | 63 | 20.7 (10.3) |
|  | ≥ 3 | 31 | 33.0 (10.8) | 23 | 34.2 (10.5) |
|  | **Previous treatment with INF beta-1b** |  |  |  |  |
|  | Yes | 97 | 23.5 (11.3) | 67 | 23.0 (10.9) |
|  | No | 34 | 24 (10.8) | 31 | 26.6 (13.3) |
|  | **Participation in BETAPLUS program** |  |  |  |  |
|  | Yes | 80 | 25.5 (11.3) | 55 | 27.7 (12.0) |
|  | No | 51 | 20.7 (10.2) | 43 | 19.5 (9.9) |
| **Total FSMC (scale: 20-100)** | | | | | |
|  | **Total** | 66 | 47.3 (21.9) | 95 | 46.1 (21.9) |
|  | **Age** |  |  |  |  |
|  | < 40 | 31 | 36.7 (15.8) | 44 | 41.3 (18.5) |
|  | ≥ 40 | 35 | 56.6 (22.5) | 51 | 50.2 (24.0) |
|  | **Gender** |  |  |  |  |
|  | Female | 46 | 48.3 (22.5) | 65 | 48.5 (22.7) |
|  | Male | 20 | 45.0 (20.9) | 30 | 41.0 (19.7) |
|  | **EDSS baseline score** |  |  |  |  |
|  | < 3 | 35 | 38.3 (17.6) | 61 | 39.5 (18.6) |
|  | ≥ 3 | 19 | 65.0 (21.3) | 23 | 64.3 (20.7) |
|  | **Previous treatment with INF beta-1b** |  |  |  |  |
|  | Yes | 44 | 47.4 (22.9) | 65 | 43.9 (20.3) |
|  | No | 22 | 47.0 (20.4) | 30 | 50.8 (25.0) |
|  | **Participation in BETAPLUS program** |  |  |  |  |
|  | Yes | 44 | 52.7 (23.0) | 53 | 52.2 (22.3) |
|  | No | 22 | 36.3 (14.8) | 42 | 38.5 (19.1) |
| **SDMT Total (scale: 0-110)** | | | | | |
|  | **Total** | 116 | 47.9 (12.6) | 75 | 51.5 (14.4) |
|  | **Age** |  |  |  |  |
|  | < 40 | 52 | 51.5 (13.0) | 33 | 57.4 (13.5) |
|  | ≥ 40 | 64 | 45.0 (11.6) | 42 | 46.8 (13.6) |
|  | **Gender** |  |  |  |  |
|  | Female | 79 | 49.1 (12.0) | 55 | 51.8 (13.5) |
|  | Male | 37 | 45.4 (13.5) | 20 | 50.7 (17.1) |
|  | **EDSS baseline score** |  |  |  |  |
|  | < 3 | 76 | 50.4 (11.8) | 51 | 54.8 (13.3) |
|  | ≥ 3 | 27 | 41.3 (13.0) | 18 | 45.8 (15.7) |
|  | **Previous treatment with INF beta-1b** |  |  |  |  |
|  | Yes | 84 | 49.0 (11.6) | 53 | 51.6 (12.3) |
|  | No | 32 | 45.0 (14.6) | 22 | 51.1 (19.0) |
|  | **Participation in BETAPLUS program** |  |  |  |  |
|  | Yes | 69 | 47.3 (12.0) | 44 | 51.4 (14.6) |
|  | No | 47 | 48.8 (13.4) | 31 | 51.6 (14.4) |

*SD* standard deviation, *EDSS* expanded disability status scale, *FAMS* Functional Assessment of Multiple Sclerosis, *HADS* Hospital Anxiety and Depression Scale, *CES-D* Center for Epidemiologic Studies Depression Scale, *SDMT* The Symbol Digit Modalities Test, *FSMC* Fatigue Scale for Motor and Cognitive Functions
