# Supplementary material for: Adherence, satisfaction and functional health status among patients with multiple sclerosis using the BETACONNECT® autoinjector: a prospective observational cohort study
Source: BMC Neurol. 2017 Sep 6;17:174. doi: 10.1186/s12883-017-0953-8 (PMC5588619; doi:10.1186/s12883-017-0953-8)
Supplement: Supplementary file 7 — Adherence, satisfaction and functional health status among patients with multiple sclerosis using the BETACONNECT® autoinjector: a prospective observational cohort study. Description of data: results on further injection related data and use of electronic features of the BETACONNECT® are provided. (DOCX 12 kb) [file 12883_2017_953_MOESM7_ESM.docx]

**Supplementary results to:**

*Kleiter I., et al.: Adherence, satisfaction and functional health status among patients with multiple sclerosis using the BETACONNECT^®^ autoinjector: a prospective observational cohort study*

*Further injection related data*

Most injections performed until week 24 were applied between 8 pm and 12 midnight (approximately 50%) and between 4 pm and 8 pm (almost 40%). For the majority of injections, patients used needle depths of 12 and 10 mm (45.2 and 37.7%, respectively) with an injection speed set to medium (51.5%) or low (32.4%).

*Use of electronic features of the BETACONNECT*

Approximately 20% of the patients used the additional electronic features (e.g. the reminder function) of the BETACONNECT^®^ (week 4: 21.6%, week 12: 18.9%, week 12: 20.2%).
